# The Relationship Between Judgments of Evil and Punishment Judgments

**DOI:** 10.1111/nyas.70054

**Published:** 2025-09-11

**Authors:** Ryan Wheat, Geoffrey Goodwin

**Affiliations:** ^1^ Department of Psychology University of Pennsylvania Philadelphia Pennsylvania USA

**Keywords:** blame, evil, moral character, moral judgment, punishment, wrongness

## Abstract

What consequences result from judging a given act (or its perpetrator) as evil? Because evil actions represent the worst possible forms of immorality, and that on some conceptions evil people are irredeemable, it stands to reason that judgments of evil would predict severe punishments. However, surprisingly little is known about precisely how judgments of evil relate to judgments of punishment. We theorized that judgments of evilness should add unique predictive value beyond comparable, and more widely studied, measures of wrongness, blame, and moral character. In a preregistered study, participants (*N* = 238) made moral judgments and punishment recommendations in response to a comprehensive range of wrongs (e.g., theft, battery, manslaughter, murder). Results revealed three general findings. First, judgments of evil uniquely predicted punishment recommendations beyond related moral judgments (e.g., wrongness, blame, moral character). Second, judgments of evil uniquely predicted death penalty endorsement and judgments of an offender's potential rehabilitation, whereas other moral judgments did not always do so. Finally, death penalty endorsement and rehabilitation judgments were better associated with person judgments than with act judgments, whereas more general punishment judgments showed no such divergence. These findings illuminate the predictive power of judgments of evil with regard to punishment judgments.

## 1 Introduction

Evil is hardly an obscure concept in everyday discourse. The term has often been invoked by political leaders, including recent US Presidents Bush, Obama, Biden, and Trump, in response to mass shootings, war crimes, and other shockingly heinous acts. These acts constitute the most extreme forms of immorality—yet, people learn about infractions of this sort occurring across the globe in daily life. And although there is little contest that such infractions are judged as wrong or immoral, little psychological research has focused on whether ordinary people also judge them as evil. This gap in research has persisted notwithstanding general interest in the concept of evil among influential social psychologists [1, 2, 3]. There is, accordingly, a need for greater research on moral judgments of evil.

Perhaps one reason for this relative lack of research is that the term evil is seen as too extreme to apply to real people. Indeed, some authors have argued that because this term is religiously or supernaturally loaded, it is not applicable to ordinary events and people [4, 5, 6]. However, what evidence does exist suggests that laypeople think otherwise. People, on average, endorse abstract statements about the existence of evil acts and persons slightly above the midpoint of the relevant scales [7, 8] and have also been shown to judge some others evil in response to concrete descriptions of their deviant behavior [9]. Moreover, when asked to evaluate severe cases of murder, evil frequently appears in people's open‐ended responses [10]. Clearly then, the concept of evil appears in lay judgments of extreme moral infractions, but precisely what role evil plays in guiding punishment decisions is not yet well understood. The purpose of this article is therefore to assess the role that judgments of evil play in determining punishment decisions in comparison to other, related moral judgments.

### 1.1 What Makes an Act Evil, and Does This Differ From What Makes an Act Morally Wrong?

What makes something “evil” has been difficult for scholars to pinpoint, especially in comparison to what makes something wrong. Some have argued that malignant psychological states such as sadistic pleasure [11], the silencing of moral considerations [12, 13], and malicious motives [14, 15] are essential to making a given act “evil.” Some of these scholars argue that such factors make evil acts more than just extreme cases of wrongdoing; evil acts are, instead, qualitatively distinct. Others, however, argue that none of these factors can explain the full range of acts people think of as evil. Rather, the only shared attribute among all evil acts is a connection to extreme harm. Given that a connection to harm is a feature of many mere wrongs, evil and ordinary wrongdoing are quantitatively, rather than qualitatively, distinct [16]. Russell's conception of evil [17] is, accordingly (p. 67):

An action is evil if and only if it is a wrong that is extremely harmful for at least one individual victim, where the wrongdoer is fully culpable for that harm in its extremity, or

it is an action that is appropriately connected to an actual or possible extreme harm of this kind, and the agent is fully culpable for that action.

Russell goes on to say that (p. 67, emphasis added):

This is intended to capture all and only the cases of the morally worst kind of wrongdoing. This may seem to be a fairly complex definition, but the general idea could be captured in the following slogan: *evil actions are extreme culpable wrongs*.

This definition, though clear and well‐defended conceptually, contains an important assumption—namely, that all evil acts are also acts of wrongdoing. Though plausible, this assumption is amenable to empirical testing, and as we describe below, it may not hold in all cases. Furthermore, any conceptual definition of evil leaves unanswered a question regarding whether distinct psychological processes underpin judgments of evil and wrongness.

In some of our own recent work, we have found evidence supporting a divergent process account [10]. In essence, although judgments of wrongness and judgments of evil are equally well predicted by people's assessments of harm (and injustice), judgments of evil are better predicted than are judgments of wrongness by people's assessments of whether the perpetrator held an unjustified disdainful attitude toward the target of the action. As a result, when people read about decisions that have exceedingly good consequences but stem from a disdainful attitude toward the welfare of those affected by the decision, they judge these acts “evil” more often than they judge them morally wrong. This finding—that some acts are more readily judged evil than morally wrong—provides evidence in support of the idea that divergent processes underlie judgments of evil and wrongness, and it also challenges the definition of evil given above. In addition to this evidence, we found that varying a given perpetrator's motives to be either disdainful or merely selfish affected judgments of evil more than it affected judgments of wrongness, again suggesting a divergence between the processes underlying these judgments. Lastly, we found that participants’ perceptions of an offender's disdainful attitudes better predicted their judgments of an act's evilness than judgments of an act's wrongness—whereas in contrast, judgments of the harm and injustice caused by the offender's actions did not differentially predict evil and wrongness judgments. In sum, a growing body of evidence is beginning to suggest that moral judgments of evil and moral judgments of wrongness have qualitatively distinct antecedents.

The present research, however, centers on whether judgments of evil and wrongness have distinct downstream consequences. Here, we focus on punishment judgments and beliefs about rehabilitative potential as two key factors that may be influenced by judgments of evil.

### 1.2 Evil and Punishment

We are not the first to connect evil with punishment. Some researchers have argued that labeling an individual evil implies that they have been entirely corrupted or overtaken by supernatural forces [4, 5], and perhaps most revealingly, that they ought to be treated as a write‐off [18]. These beliefs may in turn lead people to judge that evildoers cannot be rehabilitated and must instead be eradicated—thereby licensing especially brutal forms of punishment toward them (e.g., the death penalty). For example, if an evil person is truly a write‐off, not only should that person be punished especially harshly, but that person should also be seen as unable to be rehabilitated.

Indeed, some existing empirical work has found evidence linking lay judgments of evil to punishment. Webster et al. [8] developed a scale that captures individual differences in the *belief in pure evil* (BPE). The most general BPE item is “Some people are just pure evil.” But there are also items that capture beliefs that evil people cannot be reformed (e.g., “Evil people have an evil essence, like a stain on their souls, which is almost impossible to get rid of”) and should be eradicated from the earth (e.g., “We could obtain a more peaceful society by simply wiping out all evildoers”). Perhaps not surprisingly, scores on this scale were found to be positively associated with general support for the death penalty as well as more severe jail sentence recommendations for specific criminal offenders and were negatively associated with general support for the rehabilitation of law‐breaking individuals. These findings are clearly consistent with the idea that labeling others “evil” results in an endorsement of severe punishment.

The evidence from this study, however, consists only of abstract support for the death penalty and rehabilitation, rather than judgments about the punishment and rehabilitation prospects of a specific person who performed a specific act, raising the question of whether this pattern of results would hold for more concrete stimuli. Gromet et al. [9] provided some support that it does. Participants read a series of vignettes describing target individuals who either did or did not experience sadistic pleasure after committing a murder. They then provided judgments of the target's evilness, the likelihood that the target would reoffend, and their support for a court's decision to assign the target the death penalty. Results revealed that the target person's increasingly positive hedonic state in response to harm was associated with greater support for that person receiving the death penalty, and that this relationship was mediated by judgments of the person's evilness beyond their judged likelihood of reoffending. These findings therefore accord with the idea that judgments of evil are strongly connected to judgments of extreme punishment.

However, although this work examined the predictive power of evilness judgments in comparison with judgments of an offender's likely recidivism, it did not directly compare the predictive value of evilness judgments with that of wrongness judgments—and to the best of our knowledge, nor does any other work. Moreover, by only targeting a relatively narrow range of offenses (i.e., heinous cases of murder), it cannot establish a generalizable conclusion about how broadly judgments of evil predict punishment decisions. Consequently, it remains an open question whether, and how well, judgments of evil predict punishment decisions over and above other moral judgments and across a range of criminal offenses that vary in nature and severity.

Different views about the relationship between evil and punishment are plausible. One view is that judgments of evil are largely redundant with judgments of wrongness and moral character—and on this view, evil should not add unique predictive value in accounting for people's punishment judgments. A second view is that because judgments of evil are based on religious or supernatural ideologies that are not uniformly shared, they are unlikely to systematically track people's sense of moral disapproval. This perspective suggests that evil should not reliably predict punishment judgments in the first place. A third view, however, and the one we favor, is that judgments of evil, at both the act and person level, do indeed capture systematic variance in people's sense of moral disapproval that is not redundant with their judgments of wrongness or immorality. As such, judgments of evil are liable to predict people's punishment judgments, even when holding constant people's judgments of an act's moral wrongness or of the perpetrator's blame and moral character. The primary aim of the present study is to resolve which of these views is empirically supported.

### 1.3 Act Judgments, Person Judgments, and Punishment Judgments

A secondary aim of this study is to determine whether judgments of an offender's actions or their character better predict the punishment people subsequently assign to them. After all, some research has shown that people's judgments about a target person's moral traits are not redundant with their judgments of the morality of that person's actions [19, 20, 21, 22]. Therefore, which factor do people tend to weigh more when making punishment decisions? Immanuel Kant argued that punishment should be proportional to an offender's *inner wickedness*, without any regard to its consequences [23]. If Kant's views are taken at face value, the severity of an offender's actions should matter for punishment only insofar as they reveal the offender's malignant underlying moral character. Yet, it seems capricious to punish an offender simply for being a bad person in the absence of clear evidence that they did something illegal or immoral. Thus, an alternative perspective is that people assign punishments for specific acts of wrongdoing, rather than because of what those acts reveal about the offender's underlying character. The current study was designed in such a way to resolve this issue as well.

### 1.4 The Present Study

The present study examines the associations between evilness and punishment (i) across a comprehensive set of concrete scenarios; (ii) for distinct punishment types, including extreme punishments; and (iii) with judgments of evil pitted against other moral judgments, chiefly judgments of wrongness. It also examines whether judgments of evil uniquely predict people's views about an offender's redeemability (i.e., rehabilitative potential) and whether punishment judgments are more strongly predicted by act or person judgments. Our main prediction was that judgments of evilness would predict severe forms of punishment, particularly the death penalty, while holding judgments of wrongness constant, and that the same pattern would hold for judgments of rehabilitative potential. We were agnostic as to the role that judgments of evil would play in predicting more general forms of punishment (i.e., punishment severity, prison sentencing) as compared with judgments of wrongness.

## 2 Methods

### 2.1 Participants

The sample size was determined by the number of students who were able to enroll in the study before the end of the semester in which it was conducted (Fall 2024). Two hundred and forty‐one undergraduate students participated in exchange for course credit, although a few students were excluded from analysis for failing to complete the survey (*n* = 3). These exclusions left us with a final sample of 238 responses (*M*_Age_ = 19.76, SD_Age_ = 2.12; 82 men, 152 women, and two non‐binary individuals), which exceeded the 153 participant sample size necessary to have a power of 0.90 to detect a small effect (1% variance explained) given our design parameters. This study received approval from the University of Pennsylvania Institutional Review Board, and informed consent was obtained from all participants prior to survey administration.

### 2.2 Design, Materials, and Procedure

The study materials consisted of 20 vignettes adapted from Robinson and Kurzban ([24]; see the Supporting Information section). These vignettes describe criminal actions spanning a wide range of offense severities, including very minor offenses, such as accidentally taking another person's umbrella; more serious cases of theft, robbery, battery, manslaughter, and murder; as well as grievously malicious cases of murder. Each participant read and responded to a randomly selected set of 15 of these 20 vignettes, presented in a randomized order. We report the number of participants who responded to each vignette (Table S1) and some minor edits we made to vignette wording (see the Supporting Information section).

After reading each vignette, participants first responded to a set of questions asking them to make punishment judgments for the target individual described in the scenario. These questions were presented in a fixed order, and they concerned, first, punishment severity (“How severely should [target] be punished?” 0 = Not severely at all; 6 = Extremely severely), then prison sentencing (“What would be an appropriate prison sentence for [target]?” 1 = No sentence; 13 = Death penalty [25]), and critically, death penalty support (“How much would you support [target] receiving the death penalty?” 0 = Not at all; 6 = Extremely). A subsequent question probed participants’ beliefs that the target person in question could be rehabilitated (“Could [target] be rehabilitated, such that he is not inclined to break the law again?” 0 = Definitely no; 3 = Unclear one way or the other; 6 = Definitely yes).

Participants were then guided to a second page that displayed the same vignette in greyed‐out font. On this page, they responded to a series of questions, presented in a random order, asking them to make moral judgments based on the information provided to them. These questions probed judgments of the act's immorality (“How immoral are [target]’s actions?”), wrongness (“How morally wrong are [target]’s actions?”), and evilness (“How evil are [target]’s actions?”), as well as the target's blameworthiness (“How blameworthy is [target] for the consequences of their actions?”), immorality (“How immoral is [target] as a person?”), and evilness (“How evil is [target] as a person?”). All such judgments were made on 0 (not [judgment] at all) to 6 (extremely [judgment]) Likert‐type scale. For instance, the scale poles for the act immorality judgment read: “Not immoral at all” and “Extremely immoral.” After repeating this process for each of 15 vignettes, participants reported demographic information and received compensation.

### 2.3 Statistical Analyses

All of the main analyses were conducted in R Version 4.4.1 [26] with the *tidyverse* [27], *lme4* [28], and *lmerTest* [29] packages. VIF scores for fixed‐effects were calculated with the *car* package [30]. All analyses presented below were preregistered on aspredicted.org unless otherwise noted (https://aspredicted.org/xccd‐t3gk.pdf), and data and scripts necessary to replicate them are available on the Open Science Framework (https://osf.io/b6z24/).

## 3 Results

Because of the nested structure of these data (vignettes nested within participants), we performed a series of multilevel models to reveal associations between the relevant predictor and the outcome variables. All such models included random intercepts for participants (by‐subjects) and scenarios (by‐items) and were fitted maximally to contain the greatest number of random effects possible while still achieving model convergence. If a model failed to converge or had a singular fit, the random effects were reduced beginning with the smallest effect until the relevant issue no longer posed a problem. All predictor variables were group‐mean centered (within‐person) before analysis.

We first conducted a set of two models which (i) examined participants’ judgments of evil and wrongness as simultaneous predictors of the relevant punishments (for act judgments) and (ii) examined participants’ judgments of evil, blame, and person immorality as simultaneous predictors of the same punishments (for person judgments). In each case, the aim was to determine whether judgments of evil were uniquely associated with the punishment judgments (especially severe punishment judgments such as the death penalty) and with beliefs about the offender's prospects of rehabilitation, while holding other moral judgments constant.

Table 1 reports the unstandardized coefficients, their 95% confidence intervals (CI), and the proportion of variance across vignettes (i.e., at the vignette‐level) explained by judgments of evil over and above the other moral judgments in each model. For example, in the first act judgments model, when controlling for act evilness, a 1‐U increase in wrongness predicted a 0.27 U increase in the punishment severity participants assigned. Similarly, when controlling for act wrongness, a 1‐U increase in evilness also predicted a 0.27 increase in punishment severity. The amount of across‐vignette variance in punishment severity explained by judgments of evil (over and above judgments of wrongness) was calculated by running an unconditional model to determine the total variance in punishment attributable to vignettes (3.25) and subtracting out the unexplained across‐vignette variance after including wrongness judgments as the sole predictor of punishment (1.36). This reveals the amount of variance in punishment explained by wrongness judgments alone; dividing it by the total across‐vignette variance converts this number into a proportion‐of‐variance‐explained statistic. To subsequently determine the variance explained by judgments of act evilness over and above wrongness, this same procedure was repeated when including both judgments of wrongness and evilness as predictors of punishment severity. The difference between the proportion of variance explained for the model with evil and wrongness together (0.72) and the model with wrongness judgments alone (0.58) reveals the proportion of variance in punishment uniquely explained by evilness judgments (0.14). In other words, judgments of evil explain 14% of the variance in punishment severity decisions over and above judgments of wrongness.

**TABLE 1:** The predictive power of judgments of evil on punishment judgments and rehabilitation prospects over and above other act (act judgments) and person (person judgments) moral judgments.

Predictor	Punishment severity coefficient (95% CI)	*R* ^2^	Prison sentencing coefficient (95% CI)	*R* ^2^	Death penalty coefficient (95% CI)	*R* ^2^	Rehabilitation coefficient (95% CI)	*R* ^2^
Act judgments								
Wrongness	**0.27** (0.23, 0.30)		**0.29** (0.23, 0.34)		0.03 (−0.01, 0.08)		−0.01 (−0.07, 0.05)	
Evilness	**0.27** (0.23, 0.30)	0.14	**0.38** (0.33, 0.43)	0.13	**0.06** (0.02, 0.10)	0.07	−**0.21** (−0.26, −0.16)	0.25
Person judgments								
Blame	**0.15** (0.12, 0.18)		**0.08** (0.02, 0.13)		0.02 (−0.02, 0.06)		0.02 (−0.02, 0.06)	
Immorality	**0.22** (0.19, 0.26)		**0.29** (0.23, 0.36)		**0.05** (0.001, 0.10)		−**0.12** (−0.17, −0.07)	
Evilness	**0.19** (0.16, 0.23)	0.07	**0.38** (0.32, 0.44)	0.10	**0.09** (0.05, 0.14)	0.09	−**0.22** (−0.29, −0.16)	0.16

*Note*: The first (leftmost) column for each dependent variable reports unstandardized coefficients and 95% confidence intervals in parentheses. Bolded coefficients were significant at *p* < 0.05. The second column reports the proportion of variance across vignettes explained by judgments of evil over and above other predictors. Across all models, VIF scores did not exceed 1.99, indicating that multicollinearity was not a problem.

Across all models, when holding act wrongness constant, judgments of act evilness were significantly associated with punishment severity and prison sentencing judgments, as were judgments of act wrongness while holding act evilness constant.^1^ However, as predicted, judgments of act evilness were a significant positive predictor of death penalty support and a significant negative predictor of belief in the viability of rehabilitation—whereas judgments of act wrongness did not predict either judgment with act evilness held constant (see act judgments in Table 1). The second set of models, concentrating on the person attributes, yielded a broadly similar pattern of results to the act analyses (see Person judgments in Table 1). While holding all else constant, judgments of person evilness predicted all three punishment measures, as well as the perceived likelihood that the agent could be rehabilitated. Judgments of immorality paralleled this pattern of results, indicating that judgments of person's evilness and immorality each uniquely predicted variance in the four key dependent variables. However, although blame judgments were significantly associated with general punishment and prison sentencing, they did not significantly predict death penalty or rehabilitation judgments while controlling for the two other predictors. Across both act judgment and person judgment models, judgments of evil explained an additional 7%–25% of the variance in punishment and rehabilitation decisions over and above the set of other moral judgments.

To check the robustness of these effects, we ran a series of exploratory (i.e., non‐preregistered) linear regressions on each dependent variable for each item. These mirrored the structure of the multilevel models reported above, such that the same outcome variables were regressed onto the same predictors, thereby revealing the extent to which judgments of evil (of both acts and persons) predicted unique variance in a given punishment (or rehabilitation) judgment for each offense. Results from these analyses demonstrated that these effects were generally robust (Table S2). While holding wrongness judgments constant, judgments of act evilness uniquely predicted punishment severity in 19/20 scenarios, prison sentencing in 17/20 scenarios, death penalty support in 12/20 scenarios, and rehabilitation prospects in 10/20 scenarios. While holding blame and immorality judgments constant, judgments of person evilness uniquely predicted general punishment in 13/20 scenarios, prison sentencing in 15/20 scenarios, death penalty support in 14/20 scenarios, and rehabilitation prospects in 7/20 scenarios. Therefore, although there is some degree of heterogeneity in their effects, these first two sets of models generally find that evilness judgments—of both acts and persons—predict punishment while controlling for shared variance with judgments of act wrongness and person immorality (and blame), respectively.

We additionally ran a series of exploratory subgroup analyses to identify whether any demographic differences within our sample qualify these results. To do so, we ran 24 more models in which we interacted each predictor from each model with one of three potential demographic variables (political orientation, religiosity, and gender) for each of the four dependent variables and repeated this process for both act and person judgments as predictors (Tables S3–S5). In each of these 24 models, the main effect of judgments of evil still significantly predicted the relevant dependent variable over and above all other variables. In addition, out of 32 total interaction terms produced by these models,^2^ 25 were nonsignificant. These results suggest that our findings generally held across demographic subgroups. Regarding the seven significant interactions, two revealed that more conservative participants demonstrated a slightly more positive relationship between judgments of evil and death penalty endorsement than less conservative participants (for both act and person models). Two more revealed that more religious people tended to display a slightly less positive relationship between judgments of evil and death penalty endorsement than did less religious people (for both act and person models). The remaining three interactions found that the link between judgments of evil and punishment severity decreased for more religious participants relative to less religious participants, more conservative participants relative to less conservative participants, and non‐binary participants relative to men. Although these analyses suggest that political orientation and religiosity are important demographic variables to account for, the main effects of judgments of evil still uniformly predicted punishment and rehabilitation decisions across the board, suggesting that the broad findings of this article are robust.

The next two sets of models investigated whether punishment judgments were more strongly associated with judgments of the act or the person's character. In other words: Do people tend to punish someone for what they've done or for who they are? Because we measured both act and person judgments for immorality judgments and evilness judgments, we examined the contrast between act and person immorality judgments in one set of analyses (see immorality judgments in Table 2) and the contrast between act and person evilness judgments in another set (see evilness judgments in Table 2). We also calculated the proportion of across‐vignette variance in punishment explained by person judgments over and above act judgments alone.

**TABLE 2:** The relative predictive power of act and person moral judgments on punishment judgments and perceived rehabilitation prospects, as observed in immorality judgments and evilness judgments.

Predictor	Punishment severity coefficient (95% CI)	*R* ^2^	Prison sentencing coefficient (95% CI)	*R* ^2^	Death penalty coefficient (95% CI)	*R* ^2^	Rehabilitation coefficient (95% CI)	*R* ^2^
Immorality judgments								
Act immorality	**0.30** (0.25, 0.34)		**0.20** (0.14, 0.27)		−0.02 (−0.07, 0.03)		0.03 (−0.02, 0.08)	
Person immorality	**0.21** (0.17, 0.26)	0.08	**0.44** (0.38, 0.50)	0.10	**0.13** (0.09, 0.18)	0.11	−**0.28** (−0.35, −0.22)	0.23
Evilness judgments								
Act evilness	**0.30** (0.26, 0.34)		**0.26** (0.20, 0.32)		−0.03 (−0.08, 0.02)		−**0.05** (−0.10, −0.001)	
Person evilness	**0.16** (0.12, 0.20)	0.05	**0.40** (0.34, 0.47)	0.08	**0.15** (0.11, 0.20)	0.12	−**0.26** (−0.33, −0.19)	0.16

*Note*: The first (leftmost) column for each dependent variable reports unstandardized coefficients and 95% confidence intervals in parentheses. Bolded coefficients were significant at *p* < 0.05. The second column reports the proportion of variance across vignettes explained by person judgments over and above act judgments. Across all models, VIF scores did not exceed 2.42, indicating that multicollinearity was not a problem.

For the immorality judgments, both act and person judgments were positively associated with punishment severity and prison sentencing judgments—that is, an increase in the immorality of the act predicted an increase in punishment, controlling for the immorality of the person, and vice versa. However, regarding the endorsement of especially severe punishment (i.e., the death penalty), only person immorality was a positive predictor, whereas act immorality was not (when controlling for shared variance among predictors). The same pattern of results occurred for rehabilitation judgments, as only person immorality negatively predicted beliefs about the prospects of rehabilitation, whereas act immorality did not (see immorality judgments in Table 2). For the evilness judgments, the pattern of results was very similar but differed slightly from that for immorality judgments. Blame, person immorality, and person evilness all uniquely predicted punishment severity and prison sentencing judgments. However, only person immorality and person evilness uniquely predicted death penalty support and beliefs about rehabilitation, whereas blame did not (see evilness judgments in Table 2). Across all models, person judgments explained an additional 5%–23% of the variance in punishment and rehabilitation assessments over and above act judgments. These results generally suggest that people's attitudes toward especially severe punishments, such as the death penalty, as well as their beliefs about rehabilitation, are linked more closely to person judgments than to act judgments, whereas people's attitudes toward more general punishments are linked to act and person judgments more evenly.

To rule out vignette sequencing as a potential alternative explanation for the results presented in this article, we analyzed only the first vignette each participant responded to by running a series of non‐preregistered linear regression models utilizing the same predictors as in the previously reported analyses for each punishment outcome. The results were consistent with those reported above. Judgments of evil predicted punishment severity, prison sentencing, death penalty endorsement, and rehabilitation assessments while controlling for comparable moral judgments. Person judgments always predicted punishment decisions, whereas act judgments did not always do so. These results validate the general findings, suggesting that order effects cannot account for any of the results presented here (Table S6).

## 4 Discussion

Severe moral infractions are commonplace in daily life and are a mainstay of moral psychological research, which has investigated all manner of moral infractions. Curiously though, a commonplace epithet used to describe such acts and their perpetrators, namely, that they are “evil,” has been understudied. We currently know relatively little about people's deployment of this term, what it signifies, what it is based on, and what consequences it has. Although some recent work has focused on the antecedents of judgments of evil [10], the present work focuses on the potential consequences of such judgments—chiefly, how they predict people's punishment judgments.

Although the connection between judgments of evil and punishment has been considered in some theoretical and empirical work, it has hitherto not been examined across a comprehensive set of stimuli and for distinct kinds of punishment judgments. Furthermore, it has not previously been examined how judgments of evil differentially predict punishment in comparison with judgments of wrongness, moral character, and blame. The present study sought to address these gaps.

For act judgments, judgments of evil and judgments of wrongness both uniquely predicted general punishment attitudes when both were entered simultaneously in regression models; however, only judgments of evil uniquely predicted death penalty endorsement and reduced belief in an offender's prospects for rehabilitation, whereas judgments of wrongness did not. For person judgments, judgments of evil and person immorality uniquely predicted general and severe punishment judgments, as well as reduced belief in an offender's prospects for rehabilitation; in contrast, judgments of blame only uniquely predicted general punishment judgments. Furthermore, in a set of complementary analyses, person judgments tended to better predict punishments than did corresponding act judgments, and this was especially the case for severe punishments and for beliefs in offenders’ potential for rehabilitation. All told, the present results indicate that judgments of evil have unique predictive power beyond other, corresponding judgments of wrongness and immorality and suggest that making a judgment of evil has important downstream consequences for how people think about and determine punishment and rehabilitation.

This work therefore underscores the value of studying the folk concept of evil. Although the concept of “evil” has been criticized as unrealistic or illusory [4, 5, 6], it is nonetheless a concept that ordinary people rely upon to judge heinous acts of immorality. The fact that judgments of evil appear to predict variance in punishment decisions while controlling for shared variance in judgments of wrongness, blame, and moral character is an important and novel finding, leading us to draw two broad conclusions. First, there seems to be something unique about judgments of evil not captured by other moral judgments. This conclusion is consistent with some of our own prior work [10], which has shown that evilness judgments are distinct from wrongness judgments. Although the present work does not speak definitively as to whether there is a qualitative or merely quantitative distinction between judgments of evil and wrongness, it does reinforce the broader point that these concepts are not synonymous. Second, there appears to be a good reason to better understand how and why people make judgments of evil given that whether an individual is judged as such may meaningfully influence how others choose to punish that individual. Our stimuli were sampled from a wide range of realistic crimes [24], so it is clear that evil is not just reserved for fictional or fantastical scenarios. Instead, “evil” is used to judge serious, realistic acts and their perpetrators, which may have implications for the way juries and judges think about and determine punishment for severe offenses in states where they are asked to do so.

The finding that punishment severity and prison sentencing judgments were predicted by both act and person moral judgments, whereas death penalty endorsement and rehabilitation judgments were better predicted by person judgments than by act judgments, also has implications for moral judgment research. Advocates of person‐centered approaches to moral judgment have argued that when people judge the morality of an individual's actions, they are not so much evaluating features of the act itself as they are the features of the person behind it [31]. The present results suggest that a similar idea may extend to punishment; namely, that decisions about severe punishments are more strongly rooted in inferences about the person who perpetrated the eliciting offense than in perceptions of the offense itself. But although these results may be suggestive, they are not definitive, and future work should determine whether this is the whole story.

### 4.1 Limitations and Future Directions

One limitation of this study is that it relied upon a sample of American undergraduate students who tend to hold more liberal and less religious views than the typical American. This may limit the extent to which the study's conclusions generalize to typical jury members, who are likely to be older, more conservative, and potentially more religious than college students. However, we found that the relationship between judgments of evil and death penalty endorsements tended to be stronger for more conservative participants, suggesting that the results we observe in this article would replicate in a politically representative sample. In contrast, more religious people sometimes displayed a weaker relationship between evil and punishment than less religious people did. This may mean that a religiously representative sample could produce less robust effects than those presented in this article. This conclusion is surprising, because if “evil” possesses religious connotations, then it might seem logical to expect a tighter coupling between judgments of evil and punishment decisions. However, for practical purposes, the interactions between religiosity and judgments of evil were quite small (*b*_s_ = −0.02 to 0.03), so we suspect that, in real‐world settings, this factor would not matter greatly. Given the conflicting answers regarding the generalizability of these results to more conservative and more religious samples, future research should ideally investigate this issue further. It would be similarly worthwhile to explore the cultural generalizability of this research. For example, do judgments of evil predict punishment equally for other cultures and languages that possess different political attitudes and religious perspectives? Given the diversity of political, religious, and moral ideologies across the globe, this is a promising avenue of future research.

Another limitation of this work is that it did not take measures to circumvent social desirability bias in participants’ responses. People may be hesitant, for instance, to endorse the death penalty out of fear that their endorsement of this punishment may be viewed as inappropriate. However, to the extent that participants were responding with social desirability issues in mind, it seems unlikely to us that such responding would advantage some predictors over others. Therefore, although social desirability bias may decrease the extent to which people endorse certain punishments overall, it seems an implausible explanation for the general pattern of the results we observed.

Additionally, although the results presented in this article are consistent with the idea that labeling a wrongdoer “evil” causes people to endorse especially harsh punishment toward that person, we cannot definitively conclude that this is the direction of causality. An alternative possibility is that people assign a punishment to a given offender and then later determine their moral judgments as a post hoc justification for that punishment judgment. This explanation strikes us as somewhat unlikely because it is not clear that punishment judgments, which may require deliberation and effort (because they naturally invite comparisons to other offenses to calibrate punishment appropriately), would be more immediate than moral judgments of wrongness or evil. In addition to reverse causation, third variable explanations are also possible. For instance, people might have a globally negative reaction to acts of immorality, which in turn increases both their judgments of evil and their punishment judgments independently from one another. However, an account of this sort cannot explain why judgments of evil predict some punishment judgments (e.g., support for the death penalty) better than do judgments of wrongness and blame. Global negative reactions should seemingly impact all moral judgments roughly equally. This account also cannot explain why evil, and not wrongness, was best suited to predict judgments regarding offenders’ prospects of rehabilitation. Nevertheless, because we did not systematically manipulate any moral judgments in the present study, we cannot conclusively rule out reverse causality or third variable explanations.

Finally, the present work cannot ultimately speak to whether making judgments of evil causes punishment decisions to be biased, as some authors have argued [6], or whether instead, such decisions are defensible. The idea that using the term “evil” inculcates bias can be broken down into two different logical components. The first is that “evil” is not an accurate or realistic description of real people and their actions over possible alternatives (e.g., extremely wrong/bad). The second is that judging a given target as evil, rather than as some alternative, increases the punishment judgments people make. These two points are then connected via the argument that because judging a person as “evil” is never accurate, any increase in punishment that results from such a judgment is unwarranted. We provide some support here for the second component of this argument because we find that making a judgment of evil is associated with an increase in the punishments that people typically assign (though, as noted above, we cannot establish causality). However, the first component—the question of whether “evil” is an accurate or defensible moral judgment—is beyond the scope of the present work. We have gathered evidence elsewhere showing that people will readily make judgments of evil and endorse them as accurate descriptions of wrongdoers and their actions [10], which suggests that ordinary people do not view “evil” as an unrealistic or inappropriate judgment. This does not necessarily mean that people are correct, however, and future work should consider this evidence jointly with the current work on the relationship between “evil” and punishment.

## 5 Conclusion

The concept of evil has been largely neglected in prior work on moral judgment. Some of our recent work has focused on the antecedents of judgments of evil [10]. The current study focused on the downstream consequences of judging an act and its perpetrator as evil, namely, how such judgments predict judgments of punishment and rehabilitative potential. We reveal that when determining punishment for others, people do indeed seem to weigh how evil they and their actions are, with these considerations being especially relevant for judging that severe punishment is warranted and that the offender is irredeemable.

## Author Contributions

Ryan Wheat played an equal role in conceptualization, investigation, and methodology, as well as a lead role in data curation, formal analysis, and writing – original draft, and a supporting role in writing – review and editing. Geoffrey Goodwin played an equal role in conceptualization, investigation, and methodology, as well as a supporting role in formal analysis, and a lead role in funding acquisition, supervision, and writing – review and editing.

## Conflicts of Interest

The authors declare no conflicts of interest.

## Peer Review

The peer review history for this article is available at: https://publons.com/publon/10.1111/nyas.70054.

## Supporting information




**Supplementary Material**: nyas70054‐sup‐0001‐SuppMat.docx

## Data Availability

The data that support the findings of this study are openly available in the Open Science Framework at https://osf.io/b6z24/.

## Footnotes

1 Because ratings of act immorality were highly associated with ratings of act wrongness within‐persons (*b* = 0.85, *R* ^2^ = 0.94), and our research question focused on comparing judgments of evil and wrongness, we did not include act immorality ratings in this series of models.

2 An additional eight interaction terms resulted from models interacting gender with judgments of evil, because there were three levels (man/woman/non‐binary) for this variable.

## References

[1] R. F. Baumeister , Evil: Inside Human Violence and Cruelty, 1st ed. (Holt Paperbacks, 2001).

[2] J. M. Darley , “Social Organization for the Production of Evil,” Psychological Inquiry 3, no. 2 (1992): 199–218.

[3] P. Zimbardo , The Lucifer Effect: Understanding How Good People Turn Evil (Random House, 2007).

[4] I. Clendinnen , Reading the Holocaust (Cambridge University Press, 1999).

[5] P. Cole , The Myth of Evil (University of Edinburgh Press, 2006).

[6] J. Knoll , “The Recurrence of an Illusion: The Concept of “Evil” in Forensic Psychiatry,” Journal of the American Academy of Psychiatry and the Law 36 (2008): 105–116. 18354131

[7] B. Bastian , P. Bain , M. D. Buhrmester , et al., “Moral Vitalism: Seeing Good and Evil as Real, Agentic Forces,” Personality and Social Psychology Bulletin 41, no. 8 (2015): 1069–1081, 10.1177/0146167215589819. 26089349

[8] R. J. Webster and D. A. Saucier , “Angels and Demons Are among Us: Assessing Individual Differences in Belief in Pure Evil and Belief in Pure Good,” Personality and Social Psychology Bulletin 39, no. 11 (2013): 1455–1470, 10.1177/0146167213496282. 23885037

[9] D. M. Gromet , G. P. Goodwin , and R. A. Goodman , “Pleasure from another's Pain: The Influence of a Target's Hedonic States on Attributions of Immorality and Evil,” Personality and Social Psychology Bulletin 42, no. 8 (2016): 1077–1091, 10.1177/0146167216651408. 27277282

[10] R. Wheat and G. P. Goodwin . The Difference Between Judgments of Evil and Judgments of Wrongness [Unpublished Manuscript], (Department of Psychology, University of Pennsylvania, 2025).

[11] H. Steiner , “Calibrating Evil,” Monist 85, no. 2 (2002): 183–193.

[12] E. Garrard , “The Nature of Evil,” Philosophical Explorations 1, no. 1 (1998): 43–60, 10.1080/10001998018538689.

[13] E. Garrard , “Evil as an Explanatory Concept,” Monist 85, no. 2 (2002): 320–336.

[14] T. Calder , “Is Evil Just Very Wrong?,” Philosophical Studies: An International Journal for Philosophy in the Analytic Tradition 163, no. 1 (2013): 177–196.

[15] J. Kekes , The Roots of Evil (Cornell University Press, 2005).

[16] L. Russell , “Is Evil Action Qualitatively Distinct From Ordinary Wrongdoing?,” Australasian Journal of Philosophy 85, no. 4 (2007): 659–677, 10.1080/00048400701728566.

[17] L. Russell . “The Banality of Evil,” in Evil: A Very Short Introduction, 1st ed., ed. L. Russell (Oxford University Press, 2022), 53–69, 10.1093/actrade/9780198819271.003.0004.

[18] K. M. Fincher and P. E. Tetlock , “Brutality Under Cover of Ambiguity: Activating, Perpetuating, and Deactivating Covert Retributivism,” Personality and Social Psychology Bulletin 41, no. 5 (2015): 629–642, 10.1177/0146167215571090. 25758706

[19] D. Tannenbaum , E. L. Uhlmann , and D. Diermeier , “Moral Signals, Public Outrage, and Immaterial Harms,” Journal of Experimental Social Psychology 47, no. 6 (2011): 1249–1254, 10.1016/j.jesp.2011.05.010.

[20] E. L. Uhlmann , L. L. Zhu , and D. Tannenbaum , “When It Takes a Bad Person to Do the Right Thing,” Cognition 126, no. 2 (2013): 326–334, 10.1016/j.cognition.2012.10.005. 23142037

[21] E. L. Uhlmann , L. L. Zhu , and D. Diermeier , “When Actions Speak Volumes: The Role of Inferences About Moral Character in Outrage Over Racial Bigotry,” European Journal of Social Psychology 44, no. 1 (2014): 23–29, 10.1002/ejsp.1987.

[22] E. L. Uhlmann and L. L. Zhu , “Acts, Persons, and Intuitions: Person‐Centered Cues and Gut Reactions to Harmless Transgressions,” Social Psychological and Personality Science 5, no. 3 (2014): 279–285, 10.1177/1948550613497238.

[23] I. Kant . “The Science of Right,” In *Great Books of the Western World* , ed. R. Hutchins (W. Hastie, trans). (Edinburgh, Scotland: T. & T. Clark. 1952 [Original work published 1790]), Vol. 42, pp. 397–446.

[24] P. H. Robinson and R. Kurzban , “Concordance and Conflict in Intuitions of Justice,” Minnesota Law Review 91 (2006): 1829.

[25] J. M. Darley , K. M. Carlsmith , and P. H. Robinson , “Incapacitation and Just Deserts as Motives for Punishment,” Law and Human Behavior 24, no. 6 (2000): 659–683, 10.1023/A:1005552203727. 11105478

[26] R Core Team . R: A Language and Environment for Statistical Computing (Version 4.4.1) [Computer Software]. R Foundation for Statistical Computing (R Core Team, 2024), https://www.R‐project.org.

[27] H. Wickham , M. Averick , J. Bryan , et al., “Welcome to the Tidyverse,” Journal of Open Source Software 4, no. 43 (2019): 1686, 10.21105/joss.01686.

[28] D. Bates , M. Mächler , B. Bolker , and S. Walker , “Fitting Linear Mixed‐Effects Models Using Lme4,” Journal of Statistical Software 67, no. 1 (2015): 1–48, 10.18637/jss.v067.i01.

[29] A. Kuznetsova , P. B. Brockhoff , and R. H. B. Christensen , “LmerTest Package: Tests in Linear Mixed Effects Models,” Journal of Statistical Software 82, no. 13 (2017): 1–26, 10.18637/jss.v082.i13.

[30] J. Fox and S. Weisberg , An R Companion to Applied Regression (Sage, 2019), https://www.john‐fox.ca/Companion/.

[31] E. L. Uhlmann , D. A. Pizarro , and D. Diermeier , “A Person‐Centered Approach to Moral Judgment,” Perspectives on Psychological Science 10, no. 1 (2015): 72–81, 10.1177/1745691614556679. 25910382

