# Supplementary material for: The Relationship Between Judgments of Evil and Punishment Judgments
Source: Ann N Y Acad Sci. 2025 Sep 11;1552(1):165–73. doi: 10.1111/nyas.70054 (PMC12576864; doi:10.1111/nyas.70054)
Supplement: Supplementary file 1 — Supplementary Material: nyas70054‐sup‐0001‐SuppMat.docx [file NYAS-1552-165-s001.docx]

**TABLE OF CONTENTS**

Stimuli 2

Table S1 7

Table S2 9

Table S3 16

Table S4 17

Table S5 18

Table S6 20

Stimuli

A few changes were made to the original Robinson and Kurzban materials for use in our study. First, we wrote each vignette to describe the actions of different agents and eliminated prior misdeeds from cases of negligent manslaughter (S16 & S17). Each of these changes was made with the intention of clarifying to participants which specific actions they should judge. Additionally, we dropped four vignettes from the original materials which possessed substantial overlap with other vignette content. Minor syntax edits were made for reading clarity. The specific materials we used are reported below (vignette number refers to the number in Robinson and Kurzban’s original materials).

S2: COERCIVE THREAT TO CHILD

A man grabs Warren’s child and puts a sharp knife to her throat. He tells Warren that he will kill the child if Warren does not steal an expensive digital camera from a nearby shop, or if he attempts to contact police. Because the man can see everything he does, Warren does as he is told in order to save his child -- and steals the expensive digital camera.

S3: UMBRELLA MISTAKE

John takes another person’s umbrella assuming it to be his own because it has the same unusual color pattern as his own, a fact that the police confirm.

S5: WHOLE PIES FROM BUFFET

The owner of an all-you-can-eat buffet has posted rules that expressly prohibit taking food away; patrons can only take what they eat at the buffet. The owner has set the price of the buffet accordingly. Jesse purchases dinner at the buffet, but when he leaves he takes with him two whole pies to give to a friend.

S6: LOGO T-SHIRT FROM STORE

In a small family-owned music store, Terry notices a T-shirt with the logo of his favorite band. While the store clerk is preoccupied with inventory, Terry places the $30 T-shirt in his coat and walks out, with no intention of paying for it.

S7: SHORT CHANGE CHEAT

Derrick is a cab driver who picks up a high school student. Because the customer seems confused about the monetary transaction, Derrick decides he can trick her and gives her $30 less change than he knows she is owed.

S8: ELECTRIC DRILL FROM GARAGE

Aaron does not have all the tools he needs for his workshop but knows of a family two streets over who sometimes leave unlocked the door to the detached garage next to their house. When he next sees his chance, he enters the detached garage through the unlocked door and takes a medium-size electric drill, intending to keep it forever.

S9: SMASHING TV

While a family is away for the day, Paul breaks in through a bedroom window and rummages through the house looking for valuables. He can only find an 18-inch television, which angers him. When he gets it outside, he realizes that it is an older model than he wants, so he smashes it onto the driveway, breaking it into pieces.

S10: SLAP & BRUISING AT RECORD STORE

A record store patron is wearing a cap that mocks William's favorite band. William follows him from the store, confronts him, then slaps him in the face hard, causing him to stumble. The man’s face develops a harsh black and yellow bruise that does not go away for some time.

S11: HEAD-BUTT AT STADIUM

While attending a football game, Trent becomes angry as he overhears an opposing fan’s disparaging remarks about Trent’s team. At the end of the game, Trent sticks his face in the man’s face and head-butts him, causing a black eye and a gash that requires two stitches to close.

S12: STITCHES AFTER SOCCER GAME

Angry after overhearing another parent’s remarks during a soccer match in which Brandon’s son is playing, Brandon approaches the man after the game, grabs his coffee mug, knocks him down, then kicks him several times while he is on the ground, knocking him out for several minutes and causing cuts that require five stitches.

S13: NECKLACE SNATCH AT MALL

As a woman searches her purse for car keys in a mall parking lot, Sam runs up and grabs her gold necklace but it does not break. He yanks the woman to the ground by her necklace, causing her to gash her head, requiring stitches. Sam runs off without the necklace.

S14: ATTEMPTED ROBBERY AT GAS STATION

Owen demands money from a man buying gas at a gas station. When the man refuses, Owen punches the man several times in the face, breaking his jaw and causing several cuts that each require stitches. He then runs off without getting any money.

S15: CLUBBING DURING ROBBERY

To force a man to give up his wallet during a robbery attempt, Mike beats the man with a club until he relinquishes his wallet, which contains $350. The man must be hospitalized for two days.

S16: MAULING BY PIT BULLS

Two dogs that Kevin owns have just learned to escape, and have attacked a person who came to Kevin’s house. The police tell Kevin he must destroy the dogs, which he agrees to do, but does not intend to do. The next day, the dogs escape again and maul to death a man delivering a package.

S17: INFANT DEATH IN CAR

Randy is driving to see a man about buying a rare collector's item but must baby-sit his friend’s toddler son. It occurs to him that it is too hot to safely leave the toddler in the car, but he decides to leave him anyway and to return soon. He gets talking with the seller, however, and forgets about the toddler, who passes out and dies.

S18: STABBING

Greg is offended by a woman’s mocking remark and decides to hurt her badly. At work the next day, when no one else is around, he picks up a letter opener from his desk and stabs her. She later dies from the wound.

S19: AMBUSH SHOOTING

Jerrett knows the address of a woman who has highly offended him. As he had planned the day before, he waits there for the woman to return from work and, when she appears, Jerrett shoots her to death.

S20: ABDUCTION SHOOTING

A woman at work reveals Chris's misdeeds to his employer, thereby getting him fired. Chris devises a plan to get even with her. The next week he forces the woman into his car at knife point and drives her to a secluded area where he shoots her to death.

S21: BURNING MOTHER FOR INHERITANCE

Theo works out a plan to kill his 60-year-old invalid mother for the inheritance. He drags her to her bed, puts her in, and lights her oxygen mask with a cigarette, hoping to make it look like an accident. The elderly woman screams as her clothes catch fire and she burns to death. Theo just watches her burn.

S22: RANSOM, RAPE, TORTURE & STRANGLING

Ken kidnaps an 8 year-old girl for ransom, rapes her, then records the child’s screams as he burns her with a cigarette lighter, sending the recording to her parents to induce them to pay his ransom demand. Even though they pay as directed, Ken strangles the child to death to avoid leaving a witness.

Table S1.

*Total number of responses to each of the 20 vignettes (N = 238).*

| Vignette | # of Responses |
| --- | --- |
| S2: COERCIVE THREAT TO CHILD | 179 |
| S3: UMBRELLA MISTAKE | 178 |
| S5: WHOLE PIES FROM BUFFET | 179 |
| S6: LOGO T-SHIRT FROM STORE | 179 |
| S7: SHORT CHANGE CHEAT | 179 |
| S8: ELECTRIC DRILL FROM GARAGE | 179 |
| S9: SMASHING TV | 179 |
| S10: SLAP & BRUISING AT RECORD STORE | 179 |
| S11: HEAD-BUTT AT STADIUM | 179 |
| S12: STITCHES AFTER SOCCER GAME | 180 |
| S13: NECKLACE SNATCH AT MALL | 178 |
| S14: ATTEMPTED ROBBERY AT GAS STATION | 179 |
| S15: CLUBBING DURING ROBBERY | 179 |
| S16: MAULING BY PITBULLS | 179 |
| S17: INFANT DEATH IN CAR | 179 |
| S18: STABBING | 179 |
| S19: AMBUSH SHOOTING | 179 |
| S20: ABDUCTION SHOOTING | 180 |
| S21: BURNING MOTHER FOR INHERITANCE | 179 |
| S22: RANSOM, RAPE, TORTURE & STRANGLING | 180 |

Table S2.

*Regression analyses indicating how the various moral judgments predicted punishment and rehabilitation decisions for each individual scenario*. *Eight regression analyses are presented for each scenario, as a function of type of judgment (act vs. person) and type of punishment decision (punishment severity, prison sentencing, death penalty, rehabilitation).*

|  | Dependent Variable | | | | |
| --- | --- | --- | --- | --- | --- |
| Scenario | Predictor | Punishment  Severity | Prison Sentencing | Death Penalty | Rehabilitation |
| S2: COERCIVE THREAT TO CHILD | Act Judgments |  |  |  |  |
|  | Wrongness | .20*** | .23** | -.06^+^ | -.17 |
|  | Evilness | .52*** | .63*** | .24*** | -.12 |
|  | Person Judgments |  |  |  |  |
|  | Blame | .39*** | .56*** | -.12* | .07 |
|  | Immorality | .22^+^ | -.08 | .12 | -1.07*** |
|  | Evilness | .26^+^ | .59** | .26** | .56^+^ |
| S3: UMBRELLA MISTAKE | Act Judgments |  |  |  |  |
|  | Wrongness | .19** | .22*** | .12*** | .21 |
|  | Evilness | .54*** | .86*** | .26*** | -.51^+^ |
|  | Person Judgments |  |  |  |  |
|  | Blame | .05 | -.03 | -.01 | -.05 |
|  | Immorality | .09 | .24*** | .13** | .06 |
|  | Evilness | .48*** | .79*** | .20*** | -.37 |
| S5: WHOLE PIES FROM BUFFET | Act Judgments |  |  |  |  |
|  | Wrongness | .21*** | -.03 | -.04 | .06 |
|  | Evilness | .31*** | .24* | .12* | -.13 |
|  | Person Judgments |  |  |  |  |
|  | Blame | .03 | -.05 | -.03 | .13^+^ |
|  | Immorality | .24** | -.01 | -.04 | -.17 |
|  | Evilness | .29** | .35* | .18* | -.06 |
| S6: LOGO T-SHIRT FROM STORE | Act Judgments |  |  |  |  |
|  | Wrongness | .29*** | .06 | -.04 | .02 |
|  | Evilness | .29*** | .45*** | .12*** | -.16^+^ |
|  | Person Judgments |  |  |  |  |
|  | Blame | .09 | -.08 | -.06* | .20** |
|  | Immorality | .27*** | .16^+^ | .02 | -.01 |
|  | Evilness | .28*** | .52*** | .15*** | -.27** |
| S7: SHORT CHANGE CHEAT | Act Judgments |  |  |  |  |
|  | Wrongness | .06 | -.06 | -.03 | .04 |
|  | Evilness | .26*** | .22** | .05^+^ | -.16 |
|  | Person Judgments |  |  |  |  |
|  | Blame | .11 | -.05 | -.05^+^ | .19^+^ |
|  | Immorality | .04 | .04 | .01 | -.05 |
|  | Evilness | .28*** | .24** | .06* | -.26* |
| S8: ELECTRIC DRILL FROM GARAGE | Act Judgments |  |  |  |  |
|  | Wrongness | .28*** | .25** | -.04 | .09 |
|  | Evilness | .26*** | .35*** | .14*** | -.24* |
|  | Person Judgments |  |  |  |  |
|  | Blame | -.04 | .03 | -.10** | .17^+^ |
|  | Immorality | .32*** | .16 | -.02 | -.18 |
|  | Evilness | .19* | .33** | .18*** | -.13 |
| S9: SMASHING TV | Act Judgments |  |  |  |  |
|  | Wrongness | .42*** | .35** | -.04 | .21* |
|  | Evilness | .18** | .18^+^ | .10* | -.28*** |
|  | Person Judgments |  |  |  |  |
|  | Blame | .14^+^ | .18 | -.18*** | .21^+^ |
|  | Immorality | .27*** | .29* | .06 | -.04 |
|  | Evilness | .16* | .24* | .10* | -.23** |
| S10: SLAP & BRUISING AT RECORD STORE | Act Judgments |  |  |  |  |
|  | Wrongness | .26*** | .05 | -.05 | .14 |
|  | Evilness | .32*** | .49*** | .06* | -.28** |
|  | Person Judgments |  |  |  |  |
|  | Blame | .13^+^ | -.19^+^ | -.14*** | .38*** |
|  | Immorality | .19* | .19 | .02 | -.24* |
|  | Evilness | .26** | .45*** | .07* | -.13 |
| S11: HEAD-BUTT AT STADIUM | Act Judgments |  |  |  |  |
|  | Wrongness | .31*** | .19^+^ | -.01 | .02 |
|  | Evilness | .30*** | .34*** | .06^+^ | -.18* |
|  | Person Judgments |  |  |  |  |
|  | Blame | .11 | .03 | -.09* | .24** |
|  | Immorality | .31*** | .05 | .05 | -.10 |
|  | Evilness | .25** | .41*** | .07^+^ | -.19* |
| S12: STITCHES AFTER SOCCER GAME | Act Judgments |  |  |  |  |
|  | Wrongness | .24*** | .32** | -.02 | .25** |
|  | Evilness | .28*** | .27** | .07^+^ | -.23** |
|  | Person Judgments |  |  |  |  |
|  | Blame | .21** | .02 | -.07^+^ | .07* |
|  | Immorality | .09 | .25* | -.01 | -.03 |
|  | Evilness | .26*** | .34** | .14** | -.18^+^ |
| S13: NECKLACE SNATCH AT MALL | Act Judgments |  |  |  |  |
|  | Wrongness | .37*** | .29* | -.13* | .21^+^ |
|  | Evilness | .16* | .29* | .12* | -.30** |
|  | Person Judgments |  |  |  |  |
|  | Blame | .22** | .09 | -.08 | .21* |
|  | Immorality | .32** | .50** | .02 | -.16 |
|  | Evilness | .01 | -.01 | .08 | -.19^+^ |
| S14: ATTEMPTED ROBBERY AT GAS STATION | Act Judgments |  |  |  |  |
|  | Wrongness | .29*** | .19 | -.14* | .31** |
|  | Evilness | .35*** | .38*** | .11* | -.31*** |
|  | Person Judgments |  |  |  |  |
|  | Blame | .26 | -.08 | -.14* | .11 |
|  | Immorality | .15 | .32* | -.09 | .002 |
|  | Evilness | .22 | .31** | .19*** | -.27** |
| S15: CLUBBING DURING ROBBERY | Act Judgments |  |  |  |  |
|  | Wrongness | .45*** | .46** | -.04 | .07 |
|  | Evilness | .25*** | .26* | .04 | -.29** |
|  | Person Judgments |  |  |  |  |
|  | Blame | .33** | .14 | -.15* | .27* |
|  | Immorality | .18^+^ | .32^+^ | .12 | -.42** |
|  | Evilness | .20** | .35** | .03 | -.16 |
| S16: MAULING BY PITBULLS | Act Judgments |  |  |  |  |
|  | Wrongness | .35*** | .53*** | -.01 | -.03 |
|  | Evilness | .16* | .31* | .23*** | -.22* |
|  | Person Judgments |  |  |  |  |
|  | Blame | .36*** | .32** | -.01 | -.05 |
|  | Immorality | .24** | .32^+^ | .03 | -.02 |
|  | Evilness | .15^+^ | .43* | .25*** | -.30* |
| S17: INFANT DEATH IN CAR | Act Judgments |  |  |  |  |
|  | Wrongness | .27*** | .54** | -.12 | -.01 |
|  | Evilness | .17*** | .32** | .32*** | -.29*** |
|  | Person Judgments |  |  |  |  |
|  | Blame | .51*** | .60** | -.08 | .22^+^ |
|  | Immorality | .08 | .39* | .08 | -.30** |
|  | Evilness | .11* | .16 | .28** | -.22** |
| S18: STABBING | Act Judgments |  |  |  |  |
|  | Wrongness | .31** | .99*** | -.52* | -.32 |
|  | Evilness | .24** | .54* | .24 | .001 |
|  | Person Judgments |  |  |  |  |
|  | Blame | .07 | .14 | -.75*** | -.08 |
|  | Immorality | .21* | .62** | .20 | .04 |
|  | Evilness | .05 | .57** | .40* | -.25^+^ |
| S19: AMBUSH SHOOTING | Act Judgments |  |  |  |  |
|  | Wrongness | .54*** | 1.01** | .10 | .34 |
|  | Evilness | .16* | .44 | -.17 | -.48 |
|  | Person Judgments |  |  |  |  |
|  | Blame | .43*** | .72** | -.61* | .42^+^ |
|  | Immorality | .09 | .38 | .29 | -.36 |
|  | Evilness | .14* | .40^+^ | .37 | -.30 |
| S20: ABDUCTION SHOOTING | Act Judgments |  |  |  |  |
|  | Wrongness | .73*** | .74** | .33 | -.32 |
|  | Evilness | .15* | .64** | -.26 | .12 |
|  | Person Judgments |  |  |  |  |
|  | Blame | .53*** | .48^+^ | .34 | -.18 |
|  | Immorality | .33*** | .32 | .15 | .10 |
|  | Evilness | -.03 | .44* | .32 | -.13 |
| S21: BURNING MOTHER FOR INHERITANCE | Act Judgments |  |  |  |  |
|  | Wrongness | .23* | .68** | -1.15^+^ | -.29 |
|  | Evilness | .46*** | .67** | 1.14* | .07 |
|  | Person Judgments |  |  |  |  |
|  | Blame | .18^+^ | .37 | -.69 | -.28 |
|  | Immorality | .48*** | .67* | .28 | -.12 |
|  | Evilness | -.06 | .09 | .49 | -.19 |
| S22: RANSOM, RAPE, TORTURE & STRANGLING | Act Judgments |  |  |  |  |
|  | Wrongness | .91*** | 1.41** | -.57 | -.48 |
|  | Evilness | .18 | .39 | 1.43 | -.52 |
|  | Person Judgments |  |  |  |  |
|  | Blame | -.11 | .21 | .36 | .23 |
|  | Immorality | .81*** | .46 | -.75 | -.56 |
|  | Evilness | .03 | .35^+^ | 1.09** | -.28 |

Note: All effects are unstandardized coefficients. Because different outcomes were measured on different scales, we caution against comparing effect sizes from one form of punishment to another.

*** *p* < .001, ** *p* < .01, * *p* < .05, ^+^ *p* < .10

Table S3.

*Analyses interacting moral judgments with political orientation*.

|  | Dependent Variable | | | | |
| --- | --- | --- | --- | --- | --- |
| Predictor | | Punishment  Severity | Prison Sentencing | Death Penalty | Rehabilitation |
| Act Judgments | |  |  |  |  |
| Wrongness | | .27*** | .27*** | .03 | .01 |
| Evilness | | .27*** | .41*** | .06** | -.22*** |
| Political Orientation | | .03 | .11** | .14*** | -.07^+^ |
| Wrongness*Political | | -.01 | -.003 | -.01 | -.002 |
| Evilness*Political | | -.01 | .02 | .06*** | .01 |
| Person Judgments | |  |  |  |  |
| Blame | | .15*** | .08** | .02 | .06* |
| Immorality | | .22*** | .28*** | .05* | -.11*** |
| Evilness | | .19*** | .39*** | .09*** | -.24*** |
| Political Orientation | | .03 | .10* | .14*** | -.07^+^ |
| Blame*Political | | -.0004 | .003 | -.02* | .02^+^ |
| Immorality*Political | | .01 | .01 | .01 | -.02 |
| Evilness*Political | | -.03** | -.001 | .06*** | .01 |

Note: For political orientation, higher units correspond to more conservative political views. All effects above are unstandardized coefficients.

*** *p* < .001, ** *p* < .01, * *p* < .05, ^+^ p < .10

Table S4.

*Analyses interacting moral judgments with religiosity*.

|  | Dependent Variable | | | | |
| --- | --- | --- | --- | --- | --- |
| Predictor | | Punishment  Severity | Prison Sentencing | Death Penalty | Rehabilitation |
| Act Judgments | |  |  |  |  |
| Wrongness | | .27*** | .29*** | .03 | .01 |
| Evilness | | .27*** | .38*** | .06* | -.22*** |
| Religiosity | | .05* | .01 | -.05^+^ | .05 |
| Wrongness*Religiosity | | .01 | -.01 | -.002 | <.001 |
| Evilness*Religiosity | | -.01 | .01 | -.03** | .01 |
| Person Judgments | |  |  |  |  |
| Blame | | .15*** | .08** | .01 | .06* |
| Immorality | | .22*** | .28*** | .05* | -.11*** |
| Evilness | | .20*** | .39*** | .09*** | -.24*** |
| Religiosity | | .05* | .01 | -.05^+^ | .05 |
| Blame*Religiosity | | .02* | .02 | .01 | -.03** |
| Immorality*Religiosity | | .01 | -.02 | -.02 | .04* |
| Evilness*Religiosity | | -.02* | .01 | -.03* | -.01 |

Note: For religiosity, higher units correspond to greater religiosity. All effects above are unstandardized coefficients.

*** *p* < .001, ** *p* < .01, * *p* < .05, ^+^ p < .10

Table S5.

*Analyses interacting moral judgments with gender*.

|  | Dependent Variable | | | | |
| --- | --- | --- | --- | --- | --- |
| Predictor | | Punishment  Severity | Prison Sentencing | Death Penalty | Rehabilitation |
| Act Judgments | |  |  |  |  |
| Wrongness | | .27*** | .29*** | .02 | .01 |
| Evilness | | .28*** | .39*** | .05* | -.21*** |
| Women | | -0.05 | .04 | .24* | .08 |
| Nonbinary | | .01 | -.51 | -.25 | .86 |
| Wrongness*Women | | .01 | -.05 | .04 | -.004 |
| Wrongness*Nonbinary | | -.03 | -.01 | .01 | .04 |
| Evilness*Women | | -.01 | .06 | .03 | -.04 |
| Evilness*Nonbinary | | -.13 | -.30 | -.13 | -.12 |
| Person Judgments | |  |  |  |  |
| Blame | | .14*** | .07* | .01 | .06^+^ |
| Immorality | | .24*** | .31*** | .03 | -.12** |
| Evilness | | .18*** | .36*** | .10*** | -.22*** |
| Women | | -.05 | .04 | .24* | .07 |
| Nonbinary | | -.001 | -.44 | -.27 | .88 |
| Blame*Women | | .02 | .01 | .01 | .001 |
| Blame*Nonbinary | | .005 | .28 | .03 | .01 |
| Immorality*Women | | -.06 | -.07 | .06 | .02 |
| Immorality*Nonbinary | | .29 | -.30 | .14 | -.22 |
| Evilness*Women | | .05 | .07 | .001 | -.06 |
| Evilness*Nonbinary | | -.37* | -.12 | -.31 | .11 |

Note: All effects above are unstandardized coefficients. Men were the base level in these models against which other levels of gender were compared.

*** *p* < .001, ** *p* < .01, * *p* < .05, ^+^ p < .10

Table S6.

*Regression analyses when restricting data to the first scenario participants viewed.*

|  | Dependent Variable | | | | |
| --- | --- | --- | --- | --- | --- |
| Predictor | | Punishment Severity | Prison Sentencing | Death Penalty | Rehabilitation |
| Act Judgments | |  |  |  |  |
| Wrongness | | .43*** | .31* | -.05 | .15 |
| Evilness | | .43*** | 1.25*** | .37*** | -.42*** |
| Person Judgments | |  |  |  |  |
| Blame | | .27*** | -.18 | -.18** | .37*** |
| Immorality | | .43*** | 1.10*** | .28** | -.37** |
| Evilness | | .25*** | .76*** | .28*** | -.26* |
| Immorality | |  |  |  |  |
| Act | | .39*** | .20 | -.21** | .10 |
| Person | | .50*** | 1.53*** | .59*** | .47*** |
| Evilness | |  |  |  |  |
| Act | | .52*** | .73*** | -.03 | -.02 |
| Person | | .23** | .88*** | .45*** | -.37*** |

Note: All effects are unstandardized coefficients. Because different outcomes were measured on different scales, we caution against comparing effect sizes from one form of punishment to another.

*** *p* < .001, ** *p* < .01, * *p* < .05, ^+^ *p* < .10
